# Nanooxide/Polymer Composites with Silica@PDMS and Ceria–Zirconia–Silica@PDMS: Textural, Morphological, and Hydrophilic/Hydrophobic Features

**DOI:** 10.1186/s11671-017-1935-x

**Published:** 2017-02-27

**Authors:** Iryna Sulym, Olena Goncharuk, Dariusz Sternik, Konrad Terpilowski, Anna Derylo-Marczewska, Mykola V. Borysenko, Vladimir M. Gun’ko

**Affiliations:** 1 Chuiko Institute of Surface Chemistry, National Academy of Science of Ukraine, 17 General Naumov Street, 03164 Kiev, Ukraine; 2 Department of Physicochemistry of Solid Surface, Faculty of Chemistry, Maria Curie-Sklodowska University, M. Curie-Sklodowska Sq. 3, 20-031, Lublin, Poland; 3 Department of Interfacial Phenomena, Faculty of Chemistry, Maria Curie-Sklodowska University, M. Curie-Sklodowska Sq. 3, 20-031, Lublin, Poland

**Keywords:** Polymeric nanocomposites, Nanosilica@PDMS, CeO_2_–ZrO_2_–SiO_2_@PDMS, Morphology, Textural characteristics, Hydrophobicity

## Abstract

SiO_2_@PDMS and CeO_2_–ZrO_2_–SiO_2_@PDMS nanocomposites were prepared and studied using nitrogen adsorption–desorption, Fourier transform infrared spectroscopy (FT-IR), scanning electron microscopy (SEM), measurements of advancing and receding contact angles with water, and microcalorimetry. The pore size distributions indicate that the textural characteristics change after oxide modification by poly(dimethylsiloxane) (PDMS). Composites are characterized by mainly mesoporosity and macroporosity of aggregates of oxide nanoparticles or oxide@PDMS nanoparticles and their agglomerates. The FT-IR spectra show that PDMS molecules cover well the oxide surface, since the intensity of the band of free silanols at 3748 cm^−1^ decreases with increasing PDMS concentration and it is absent in the IR spectrum at *C*_PDMS_ ≥ 20 wt% that occurs due to the hydrogen bonding of the PDMS molecules to the surface hydroxyls. SEM images reveal that the inter-particle voids are gradually filled and aggregates are re-arranged and increase from 20 to 200 nm in size with the increasing polymer concentration. The highest hydrophobicity (contact angle *θ* = 140° at *C*_PDMS_ = 20–40 wt%) is obtained for the CeO_2_–ZrO_2_–SiO_2_@PDMS nanocomposites. The heat of composite immersion in water shows a tendency to decrease with increasing PDMS concentration.

## Background

In recent decades, hydrophobic hybrid metal or metalloid oxide (MO)–polymer composites are widely used in a variety of applications such as self-cleaning hydrophobic coatings [1–3], chemical separation of polar and nonpolar substances [4, 5], adsorption of organic contaminants, and removal of oil from the water surface [6, 7]. The main attention is paid to development of film coatings and membranes [1–5, 8–12], as well as to highly dispersed materials with developed surface area [13–16]. To prepare highly dispersed hydrophobic composites, silica [13–15], titania [16, 17], zinc oxide [18], magnetic nanoparticles (γ-Fe_2_O_3_) [19], and mixed oxides [20–22] are often used. Highly dispersed composites with an adsorbed polymer layer on a nanoparticle surface are referred to core–shell nanocomposites (NC) [23] having a number of peculiarities. In NC with MO/poly(dimethylsiloxane) (PDMS) at a low polymer content, a major fraction of the polymer is located at the interfaces with nanoparticles [24–26]. The interfacial polymer fraction [25, 27] is characterized by a modified structure [28, 29], slower dynamics [30–33], and increased thermal stability [21] in comparison to the bulk. The use of complex MO cannot only improve the performance characteristics of the NC (such as thermal stability, durability [20, 21]) but also significantly affect their structural characteristics [20, 34]. The structure of the polymer adsorption layer defines such NC surface properties as hydrophilicity–hydrophobicity [34–36], compatibility with organics, adsorption capability, and reactivity [37–39]. The helix shape of PDMS with six Si–O bonds in a cycle [40] restricts the number of segments which can directly interact with a solid surface to form the hydrogen bonds SiO–H···O(Si(CH_3_)_2_–)_2_. However, PDMS conformation can be changed in an adsorption layer depending on the PDMS content and MO structure (i.e., cores in the core–shell particles). A variation in the PDMS content can affect many properties of NC. Thus, such PDMS/zirconia/silica characteristics as the texture, hydrophobicity, and interfacial behavior strongly differ from those of PDMS/silica [19]. Therefore, the effects of structure of complex MO, as well as the impact of the content and molecular weight of PDMS, on the structural and hydrophobic properties of core–shell NC are highly relevant for further development and enhancement of core–shell NC with improved characteristics and regulated properties.

Upon creation of NC with MO/PDMS, a particular interest should be paid to complex MO, in which each component can enhance certain properties of the whole composite. Since the presence of such oxides as CeO_2_ and ZrO_2_ in MO/PDMS increases the thermal stability [21, 34, 41], the NC based on CeO_2_–ZrO_2_–SiO_2_ could be a promising material. Preparation of similar complex nanooxides with a high specific surface was previously described in detail [42, 43].

Since the creation of the composites is primarily intended to obtain hydrophobic materials, the most important task is complete and correct characterization of their hydrophobic properties vs. structures. The hydrophobic properties of fine materials could be determined not only by measuring the contact angles [44, 45] but also by using a calorimetric technique to determine the heat of immersion in polar and nonpolar liquids [46, 47], and this method is optimal to evaluate the hydrophobicity of powder materials.

Thus, in the current study, the main attention is paid to the textural, morphological, and hydrophilic/hydrophobic properties of the polymer SiO_2_@PDMS and CeO_2_–ZrO_2_–SiO_2_@PDMS composites analyzed using SEM, adsorption, spectral, contact angle, and calorimetry methods.

## Methods

### Materials

Fumed silica (SiO_2_) (pilot plant of the Chuiko Institute of Surface Chemistry, Kalush, Ukraine) and CeO_2_–ZrO_2_/SiO_2_ were used as substrates for adsorption modification by poly(dimethylsiloxane). Silica-supported ceria–zirconia nanocomposites were prepared using a liquid-phase method and subjected to thermal treatment at 550 °C for 1 h. The content of grafted CeO_2_ was 3 and 10 wt%, and the ZrO_2_ content was constant at 10 wt% (CZS1 and CZS2, respectively). The synthesis and physicochemical characteristics of CeO_2_–ZrO_2_–SiO_2_ nanooxides were described in detail previously [42, 43].

Commercial liquid poly(dimethylsiloxane) PDMS-400 (“Kremniypolimer”, Zaporozhye, Ukraine, linear, –CH_3_ terminated; molecular weight *W*_m_ ≈ 5700, degree of polymerization *d*_p_ = 75) was adsorbed onto silica and ceria–zirconia–silica to prepare samples containing 5, 10, 20, and 40 wt% of the polymer. Before the adsorption, oxide samples were dried at 110 °C for 1 h, and then a solution of PDMS (1 wt%) in hexane was added and the suspension was stirred. The suspension was dried at room temperature for 24 h and then at 80 °C for 3 h. All samples with PDMS were in the powder form similar to the initial silica. The composition and textural characteristics of the NC are listed in Table 1.

**Table 1:** Textural characteristics of initial oxides and oxide@PDMS nanocomposites

Sample	*S*_BET_ (m^2^/g)	*S*_micro_ (m^2^/g)	*S*_meso_ (m^2^/g)	*S*_macro_ (m^2^/g)	*V*_micro_ (cm^3^/g)	*V*_meso_ (cm^3^/g)	*V*_macro_ (cm^3^/g)	*V*_p_ (cm^3^/g)	*R*_p,V_ (nm)
SiO_2_	283.4	21.0	224.9	37.5	0.008	0.348	0.569	0.925	29
SiO_2_@P5	224.8	27.1	131.9	65.8	0.002	0.054	2.168	2.224	29
SiO_2_@P10	184.4	6.2	117.3	60.9	0.004	0.612	1.250	1.866	28
SiO_2_@P20	149.2	4.1	85.6	59.5	0.003	0.638	1.152	1.793	27
SiO_2_@P40	73.9	2.2	49.6	22.1	0.000	0.027	0.925	0.952	36
CZS1	250.8	23.2	146.7	80.9	0.007	0.067	1.257	1.331	54
CZS1@P5	204.8	32.0	139.8	33.0	0.018	0.678	0.703	1.399	26
CZS1@P10	165.0	2.7	117.6	44.7	0.000	0.040	1.290	1.330	29
CZS1@P20	130.6	4.2	97.6	28.8	0.003	0.522	0.585	1.110	27
CZS1@P40	63.0	4.1	26.4	32.5	0.002	0.080	0.668	0.75	40
CZS2	258.9	14.2	158.3	86.4	0.004	0.073	1.353	1.430	55
CZS2@P5	201.3	30.2	144.6	26.5	0.017	0.729	0.549	1.295	23
CZS2@P10	156.2	19.2	123.8	13.2	0.004	0.357	0.557	0.918	22
CZS2@P20	119.8	4.7	42.9	72.2	0.006	0.561	0.330	0.897	38
CZS2@P40	57.9	6.8	35.9	15.2	0.001	0.048	0.333	0.382	28

Specific surface area in total (*S*_BET_) and of nanopores (*S*_micro_), mesopores (*S*_meso_), macropores (*S*_macro_), and respective specific pore volumes (*V*_p_, *V*_micro_, *V*_meso_, *V*_macro_). *R*_p,V_ represents the average pore radius determined from the differential pore size distributions with respect to the pore volume

### Textural Characterization

To analyze the textural characteristics of initial oxides and oxide@PDMS nanocomposites, low-temperature (77.4 K) nitrogen adsorption–desorption isotherms were recorded using an ASAP 2405N (Micromeritics Instrument Corp., USA) adsorption analyzer after outgassing the samples at 110 °C for 2 h in a vacuum chamber. The values of the specific surface area (*S*_BET_) were calculated according to the standard BET method [48]. The total pore volume *V*_p_ was evaluated from nitrogen adsorption at *p/p*_0_ = 0.98–99 (*p* and *p*_*0*_ denote the equilibrium and saturation pressures of nitrogen at 77.4 K, respectively). The nitrogen desorption data were used to compute the pore size distributions (PSD, differential *f*_V_~d*V*_p_/d*R* and *f*_S_~d*S*/d*R*) using a self-consistent regularization (SCR) procedure under nonnegativity condition (*f*_V_ ≥ 0 at any pore radius *R*) at a fixed regularization parameter *α* = 0.01 with voids (*V*) between spherical nonporous nanoparticles packed in random aggregates (V/SCR model) [49]. The differential PSD with respect to the pore volume *f*_V_~d*V*/d*R*, ∫*f*_V_d*R*~*V*_p_ were re-calculated to the incremental PSD (IPSD) at Φ_V_(*R*_*i*_) = (*f*_V_(*R*_*i*+1_) + *f*_V_(*R*_*i*_))(*R*_*i*+1_ − *R*_*i*_)/2 at ∑Φ_V_(*R*_*i*_) = *V*_p_. The *f*_V_ and *f*_S_ functions were also used to calculate contributions of micropores (*V*_micro_ and *S*_micro_ at 0.35 nm < *R* < 1 nm), mesopores (*V*_meso_ and *S*_meso_ at 1 nm < *R* < 25 nm), and macropores (*V*_macro_ and *S*_macro_ at 25 nm < *R* < 100 nm).

### Fourier Transform Infrared Spectroscopy (FT-IR)

FT-IR spectra of powdered samples (ground with dry KBr at the mass ratio 1:9) were recorded over the 4000–400 cm^−1^ range using a ThermoNicolet FT-IR spectrometer with a diffuse reflectance mode. For quantitative analysis, some IR spectra were normalized using the intensity of the Si–O vibration overtone at 1865 cm^−1^ as an inner standard.

### Scanning Electron Microscopy (SEM)

The particulate morphology was analyzed using field emission scanning electron microscopy employing a QuantaTM 3D FEG (FEI Company, USA) apparatus operating at the voltage of 30 kV.

### Contact Angle Measurements

A Digidrop GBX Contact Angle Meter (France) equipped with a video camera and firm software was used in the contact angle (CA, *θ*) measurements. Using the tilting plate method, the contact angles were measured on the pressed (180 bars for 15 min) plates of samples inclined toward the optical axis and in that position liquid gathered on one side of the droplet and retracted on the other one. A 6-μl droplet [50] was set in a chamber in front of the Contact Angle Apparatus camera and then using a small table. The droplet was tilted at an appropriate angle. The whole process was filmed until it started to slide. The advancing contact angle (ACA) was measured just before the droplet sliding on the droplet front and the receding (RCA) on its rear. The values of CA were evaluated for both sides of the sample plate using the Win Drop++ program. To obtain the averaged CA values, the measurements were performed for 10 water droplets put on each sample. The measurements were conducted at 20 °C and humidity RH = 50%.

### Apparent Surface Free Energy Determination

The apparent surface free energy of given surfaces was calculated using the contact angle hysteresis (CAH):

1 $$ {\gamma}_s=\frac{\gamma_L{\left(1+ \cos {\theta}_a\right)}^2}{2+ \cos {\theta}_r+ \cos {\theta}_a} $$

where *γ*_S_ is the apparent surface free energy, *γ*_L_ is the water surface tension (72.8 mJ/m^2^ at 20 °C), *θ*_a_ is the advancing contact angle, and *θ*_r_ is the receding contact angle of water [51].

### Microcalorimetry

Microcalorimetric investigation of oxides and composites was carried out using a DAC1.1A (Chernogolovka, Russia) differential automatic calorimeter. Before the measurements of the heat of immersion of samples in polar (water) or nonpolar (*n*-decane) liquids, the samples were degassed at 130 °C and 0.01 Pa for 2 h and then used (100 ± 5 mg per 3 ml of wetting liquid) without contact with air. Since the total heat of immersion depends on the mass and surface area of a sample, the obtained results were normalized to both 1 g of a sample and 1 m^2^ of the surface area. To obtain the averaged *Q* values, the measurements were performed two to three times for each system and the average errors were ≤7%.

## Results and Discussion

### Textural Characteristics

**Fig. 1:**
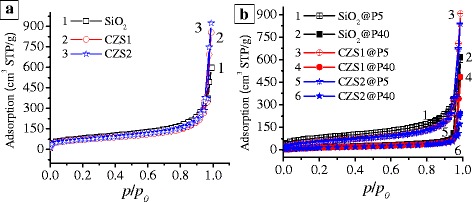
Nitrogen adsorption–desorption isotherms of oxides **a** before and **b** after adsorption of PDMS

The nitrogen adsorption–desorption isotherms for the initial oxides and composites (Fig. 1) display a sigmoidal-shaped course (type II of the IUPAC classification) with a narrow hysteresis loop of the H3 type [48, 52] in the *p*/*p*_0_ range between 0.8 and 1.0. This indicates the formation of aggregates with the initially nonporous nanoparticles that are characterized by the textural porosity.

**Fig. 2:**
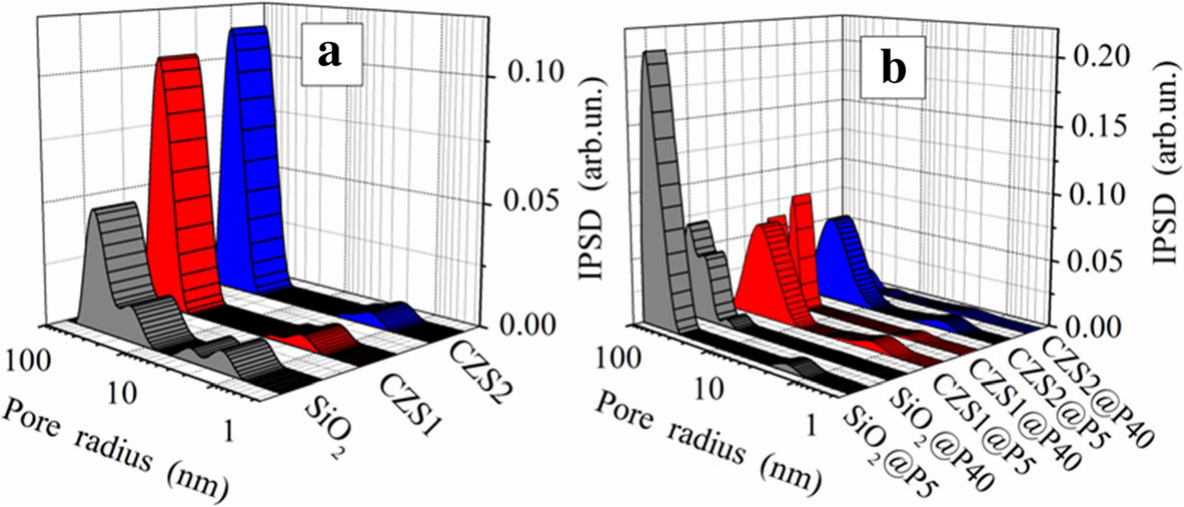
Incremental pore size distributions of oxides **a** before and **b** after adsorption of PDMS

The incremental pore (voids between nonporous nanoparticles in aggregates) size distribution functions (Fig. 2) show that the textural characteristics change after the silica modification and the formation of polymeric–nanooxide composites.

The specific surface area (Table 1, *S*_BET_) does not demonstrate a significant reduction after grafting of ceria–zirconia. All oxide samples (Fig. 2a) are mainly mesoporous (aggregates of SiO_2_ nanoparticles) and macroporous (aggregates of modified SiO_2_). In general, the average pore radii (Table 1, *R*_p,V_) are by almost twice larger in CZS1 and CZS2, as compared to unmodified silica. The textural characteristics of the materials change due to the adsorption of PDMS (Table 1 and Fig. 2b). The values of *S*_BET_ of all composites decreased with the increasing polymer content by 74, 75, and 78% (in comparison to the initial oxides) for SiO_2_@P40, CZS1@P40, and CZS2@P40, respectively, after the PDMS adsorption in the amount of 40 wt% (Table 1). The polymer adsorption leads to suppression of the pore volume (*V*_p_) as well as *V*_meso_ and *V*_macro_. After addition of PDMS, the average pore radii (Table 1, *R*_p,V_) decrease. In general, the addition of polymer can change the porosity characteristics because each long macromolecule can be able to bind several oxide nanoparticles and their aggregates in more compacted structures. This leads to the decrease in the volume of voids between particles [53].

### Fourier Transform Infrared Spectroscopy (FT-IR)

Adsorption modification of initial silica and CeO_2_–ZrO_2_–SiO_2_ nanooxides was conducted with poly (dimethylsiloxane) due to the formation of hydrogen bonds ≡ M_i_O–H···O(Si(CH_3_)_2_–)_2_ (where M = Si, Ce, or Zr, *i* = 1 or 2) between the oxygen atoms in the PDMS polymer chain and hydrogen atoms of the surface hydroxyls (–OH) of the oxide particles.

**Fig. 3:**
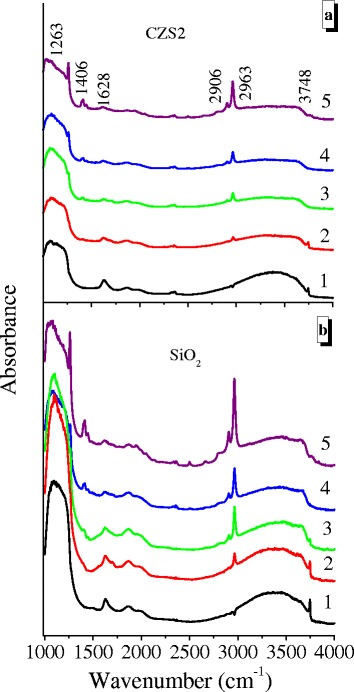
FT-IR spectra of initial oxides and oxide@PDMS nanocomposites for (**a**, curve 1) CZS2 and (**b**, curve 1) SiO_2_ at *C*_PDMS_ 5 wt% (**a**, **b** curves 2), 10 wt% (**a**, **b** curves 3), 20 wt% (**a**, **b** curves 4), and 40 wt% (**a, b** curves 5)

Figure 3 shows the FT-IR spectra in the region from 1000 to 4000 cm^−1^ for the initial oxides and oxide@PDMS composites. The broad band of high intensity at 3050–3700 cm^−1^ centered at 3300 cm^−1^ corresponds to the O–H stretching vibrations of surface hydroxyls and adsorbed water [54]. The peak located at 1628 cm^−1^ represents the bending vibrations of adsorbed water [55]. The FT-IR spectra intensity in the region of O–H vibrations gradually decreases with polymer adsorption. This suggests that a significant amount of the surface hydroxyls was disturbed by the PDMS chains and the amount of adsorbed water decreases. The FT-IR spectra show that for SiO_2_@P20 and CZS2@P20 (Fig. 3a, b, curves 4), the band of the OH-stretching vibrations of free silanols at *ν*_OH_ = 3748 cm^−1^ does not occur.

The symmetric and asymmetric C−H stretching vibrations of the methyl groups of PDMS are observed at 2906 and 2963 cm^−1^ (Fig. 3), respectively, along with the deformation vibrations of the same groups at 1263 and 1406 cm^−1^ [56]. The peak intensity depends monotonically on the amount of adsorbed polymer.

### Scanning Electron Microscopy

**Fig. 4:**
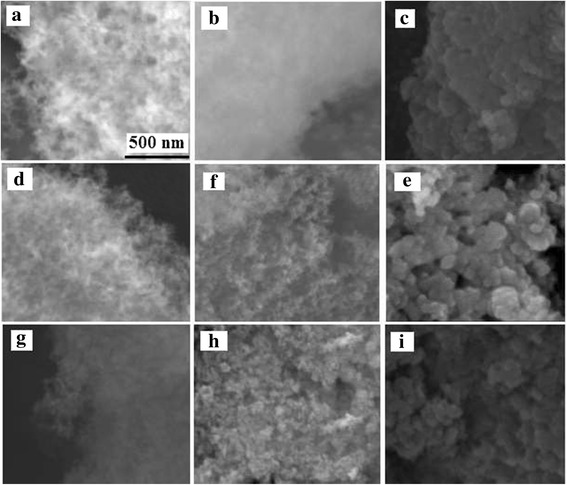
SEM images (the *scale bar* is 500 nm) of the initial oxides and oxide@PDMS nanocomposites. **a** SiO_2_. **b** SiO_2_@P5. **c** SiO_2_@P40. **d** CZS1. **f** CZS1@P5. **e** CZS1@P40. **g** CZS2. **h** CZS2@P5. **i** CZS2@P40

SEM images (Fig. 4) show changes in the outer surfaces of samples due to the adsorption of PDMS. For the composites with PDMS, a significant increase in aggregate sizes is observed, but structure of composites remained dispersed even at *C*_PDMS_ = 40 wt%. A tendency to increase the size of aggregates with the increasing PDMS concentration takes place for all samples. The structure of the initial oxides corresponds to small aggregates with a disordered loose structure. Thus, for SiO_2_, CZS1, and CZS2, the size of aggregates is 10–20 nm; upon their modification with 5 wt% PDMS, the aggregate size became from 20 to 40 nm; and when the amount of PDMS is 40 wt%, the aggregate size increases to 70–200 nm. For all composites, the polymer forms a shell of nanoparticles. The remained powder texture of oxide@PDMS composites similar to that of the initial oxide powders suggests well distributed PDMS molecules at the surfaces of all nanoparticles. A uniform coating of the oxide particles by PDMS layer occurs due to stronger interactions of PDMS with the oxide surface than with the other polymer macromolecules.

Additionally, according to [34], the average thickness of a PDMS layer was calculated. The obtained results (Table 2) show that this value depends weakly on the type of oxide composition studied. It depends mainly on the amount of PDMS. This weak influence of the particle composition is due to a relatively low content of CeO_2_ and ZrO_2_, i.e., the particulate morphology is mainly determined by the silica matrix. Taking into account aggregation of nanoparticles, one can assume that these values of *h* are minimal of possible ones. In other words, the maximal thickness of the PDMS layer could be larger, at least, by a factor of 1.3–1.5 than the average one. However, a contact zone between adjacent oxide nanoparticles in aggregates could be free of PDMS or covered by a monomolecular layer independent of the total amounts of PDMS.

**Table 2:** Average diameter (*d*) of uncoated and coated nanoparticles and average thickness (*h*) of the PDMS layer

Sample	*d* (nm)	*h* (nm)
SiO_2_	9.62	
SiO_2_@P10	15.7	3.0
SiO_2_@P40	47.6	19.0
CZS1	8.87	
CZS1@P10	14.4	2.8
CZS1@P40	47.5	19.3
CZS2	7.85	
CZS2@P10	13.5	2.8
CZS2@P40	46.8	19.5

### Hydrophilic/Hydrophobic Properties of Composites

Surface wettability is one of the research hotspots in surface science. Lately, hydrophobic surfaces, especially superhydrophobic surfaces, have attracted considerable attention because of their extensive applications in self-cleaning, biomaterials, droplet transportation, etc.

Evaluation of the hydrophobic properties of the composites was carried out by measurements of the contact angle of wetting with water. The apparent surface free energy (*γ*_S_) was determined using the ACA/RCA hysteresis approach [51].

**Fig. 5:**
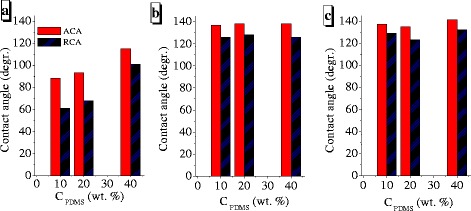
Contact angle measurements on the surface of **a** SiO_2_@PDMS, **b** CZS1@PDMS, and **c** CZS2@PDMS nanocomposites

The water contact angle measurement demonstrated that the hybrid CeO_2_–ZrO_2_–SiO_2_@PDMS surfaces (Fig. 5b, c) are much more hydrophobic than SiO_2_@PDMS (Fig. 5a) even at small amounts (10 wt%) of the polymer. However, all NC are hydrophilic at *C*_PDMS_ ≤ 5 wt%. The values of ACA and RCA depend on the nature of the oxide matrix and polymer concentration.

**Fig. 6:**
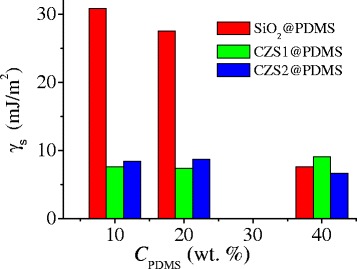
Apparent surface free energy calculated from the hysteresis (CAH) approach

The surface free energies are significantly higher for SiO_2_@PDMS than for CeO_2_–ZrO_2_–SiO_2_@PDMS composites (Fig. 6). It was found that the surface free energies extremely decreased from 30.8 ± 3.2 mJ/m^2^ to 7.6 ± 1.3 mJ/m^2^ for the SiO_2_@PDMS composites with the increasing amount of PDMS while for the CZS samples, this value hardly changes. Indeed, stronger changes in the interaction of composites with water occur before the PDMS monolayer formation because the interactions of oxide surface (hydrophilic) and PDMS (hydrophobic) with water differ largely.

### Interactions of Composites with Polar (Water) and Nonpolar (Decane) Liquids

Conditions of interactions of water with composites in the microcalorimetry method differ from those measuring the CA by a lack of the gas phase, which affects the wetting. The Gibbs free energy of the system decreases upon immersion of a composite into a liquid. This occurs due to compensating high surface energy of a solid surface interacting with a liquid that is accompanied by the heat release [57].

According to the molecular theory of wetting [58], interfacial energy is considered as a measure of the balance of dispersive and polar intermolecular interactions, and the contributions of these interactions are additive. Depending on the nature of the solid surface and liquid interacting with it, the relationship between the polar and nonpolar interactions will determine the heat of immersion [59, 60].

The heats of immersion in water (*Q*_w_) and decane (*Q*_d_) calculated per 1 g of SiO_2_@PDMS and CeO_2_–ZrO_2_–SiO_2_@PDMS nanocomposites at different *C*_PDMS_ are given in Table 3. Comparison of the heats of immersion of the oxides without PDMS showed that the modification of the SiO_2_ with Ce- and Zr-oxides results in increasing the heat of immersion in water, while the heats of immersion in decane are almost unchanged (Table 3). This indicates that CeO_2_–ZrO_2_ can enhance the surface hydrophilicity due to the formation of new active centers of the hydrophilic nature such as OH groups and bridge M–O(H)–M bonds.

**Table 3:** The heat of immersion in water and decane liquids for the SiO_2_@PDMS and CeO_2_–ZrO_2_–SiO_2_@PDMS composites

*C*_PDMS_ (wt%)	SiO_2_		CZS1		CZS2	
	*Q*_w_(J/g)	*Q*_d_(J/g)	*Q*_w_(J/g)	*Q*_d_(J/g)	*Q*_w_(J/g)	*Q*_d_(J/g)
0	41.2	20.5	57.7	22.3	60.2	19.5
5	28.1	13.4	33.9	10.8	35.9	11.6
10	13.3	5.1	11.8	7.3	13.1	7.9
20	11.9	6.1	10.7	6.1	11.5	5.3
40	3.0	2.2	6.8	4.2	8.7	1.8

After modification of nanooxides with PDMS the surface hydrophilic active centers forming the hydrogen bonds with PDMS become less accessible or inaccessible for water molecules. For all composites SiO_2_@PDMS and CeO_2_–ZrO_2_–SiO_2_@PDMS, the heats of immersion in water significantly decrease with the increasing PDMS content. In general, the heat of immersion in water is determined by the total value of wetted composite surface, the structure of adsorbed PDMS surface layer and accessibility of oxide surface for interactions with the wetting liquid. Therefore, the decrease in the heat of immersion in water can be attributed to two factors: (i) reduction of specific surface of composites with the increasing PDMS content, and (ii) changes in the structure of the surface layer interacting with water.

**Fig. 7:**
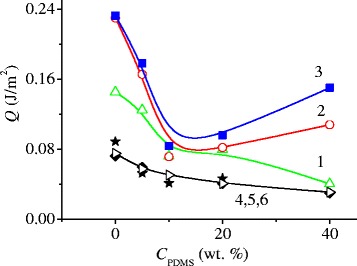
The dependence of the heat of immersion in water (*1*, *2*, *3*) and decane (*4*, *5*, *6*), normalized to 1 m^2^, on the content of PDMS composites: SiO_2_@PDMS (*1*, *4*), CZS1@PDMS (*2*, *5*), and CZS2@PDMS (*3*, *6*)

To exclude the effect of specific surface area and to determine precisely the contribution of the surface structure to the polar and nonpolar interactions, the heats of immersion in water and decane were re-calculated per 1 m^2^ (Fig. 7). In the range of PDMS concentrations prior to the formation of a monolayer on the CeO_2_–ZrO_2_–SiO_2_ surface (10–15 wt%), a sharp decrease in the immersion heat in water is observed with the increasing PDMS content. However, with a further increase in the content of PDMS, some increase in the heat of immersion in water was manifested, though CA displayed high values (Fig. 5) that defined these samples as hydrophobic. The heats of immersion in decane correlated with the values of the specific surface area; therefore, their increase for the samples with a high PDMS content was not observed (Fig. 7, Table 1). This peculiarity can be explained by the fact that the structure of the adsorbed layer strongly depends on the content of PDMS in the composite. Since during adsorption the polymers are always associated and form supramolecular structures, the structure of the adsorbed layer depends on the polymer content in the system: with the increasing polymer content PDMS macromolecules form the primary and secondary supramolecular structures, for which bonds with the solid surface may be weaker than that for flat-adsorbed molecules. At a low content, PDMS forms the adsorption layer with a flat structure, in which a maximal interaction of macromolecules with the surface active sites is implemented, and the water molecules do not have access to interact with hydrophilic active centers of nanooxides. While at a high content of PDMS, macromolecules form loops, tails, and assemblies with each other and such polymer structures can be more mobile than the flat ones during the contact with water access to the interactions of water molecules with the surface active centers of complex oxides without air absence and water excess because interaction of water with PDMS is thermodynamically unfavorable. Thus, the wetting of the composites surface depends on the structure of the adsorption layer of PDMS which changes with the increasing content of PDMS and the polar or nonpolar nature of the wetting fluid.

## Conclusions

Nanooxides (SiO_2_ and CeO_2_–ZrO_2_/SiO_2_) were used as substrates for adsorption modification by PDMS-400 in the amounts of 5, 10, 20, and 40 wt%. Effects of nanostructured oxides on the textural and morphological characteristics of polymer silica@PDMS and ceria–zirconia–silica@PDMS NC were studied employing structure (adsorption–desorption nitrogen isotherms, FT-IR) and morphology (SEM) techniques. Microcalorimetry and measurements of the contact angle of wetting with water were performed to investigate the hydrophobic properties of the materials.

It was found that the specific surface area of nanooxide@PDMS composites decreases with the increasing *C*_PDMS_, and this decrease is larger for the CZS@PDMS systems. In general, the polymer adsorption leads to decrease in the values of *V*_p_, *V*_meso_, and *V*_macro_ as well as *R*_p,V_. The analysis of the FT-IR spectra shows that all surface OH groups of oxides are disturbed due to the interactions with PMDS and adsorbed water.

The SEM images showed that the composites retain in the disperse state at the concentration of 5–40 wt% PDMS, and they can be considered as core–shell nanocomposites due to the well-distributed PDMS molecules at the surfaces of all nanoparticles of oxides.

The nanocomposites displayed high values of the main parameters related to the hydrophobicity (ACA and RCA). The contact angles of water drops for the complex oxide with adsorbed PDMS are about 140° at *C*_PDMS_ = 10–40 wt% while for the SiO_2_@PDMS composites it is of 120°. It was found that the surface free energies extremely decreased from 30.8 ± 3.2 mJ/m^2^ to 7.6 ± 1.3 mJ/m^2^ for SiO_2_@PDMS composites with the increasing amount of PDMS, while for ceria–zirconia–silica systems, this value is low (≤9.1 ± 1.3 mJ/m^2^) at any content of PDMS.

The heats of immersion in water significantly decrease with increasing PDMS content in the composites due to two factors: formation of the hydrogen bonds between the hydrophilic active sites of oxides and PDMS leading to the decrease in their accessibility to the interactions with the water molecules and reduction of the specific surface area of composite with the increasing PDMS concentration. However, at a high content of polymer (40 wt%), the interactions of composites with water can increase due to higher aggregation of PDMS molecules each other resulting to the adsorbed layer structure changes.

Thus, this study presents promising results for an easy and cost-effective alternative using such composites as protective coatings with appropriate hydrophobic properties.
